# A pilot study to demonstrate the paracrine effect of equine, adult allogenic mesenchymal stem cells *in vitro*, with a potential for healing of experimentally-created, equine thoracic wounds *in vivo*

**DOI:** 10.3389/fvets.2022.1011905

**Published:** 2022-11-14

**Authors:** Michael Caruso, Shannon Shuttle, Lisa Amelse, Hoda Elkhenany, James Schumacher, Madhu S. Dhar

**Affiliations:** ^1^Department of Large Animal Clinical Sciences, College of Veterinary Medicine, University of Tennessee, Knoxville, Knoxville, TN, United States; ^2^Department of Surgery, Faculty of Veterinary Medicine, Alexandria University, Alexandria, Egypt

**Keywords:** horse, mesenchymal stem cells, wound healing, bone marrow, allogenic cells, thoracic wounds

## Abstract

Regenerative biological therapies using mesenchymal stem cells (MSCs) are being studied and used extensively in equine veterinary medicine. One of the important properties of MSCs is the cells' reparative effect, which is brought about by paracrine signaling, which results in the release of biologically active molecules, which in turn, can affect cellular migration and proliferation, thus a huge potential in wound healing. The objective of the current study was to demonstrate the *in vitro* and *in vivo* potentials of equine allogenic bone marrow-derived MSCs for wound healing. Equine bone marrow-derived MSCs from one allogenic donor horse were used. Equine MSCs were previously characterized for their *in vitro* proliferation, expression of cluster-of-differentiation markers, and trilineage differentiation. MSCs were first evaluated for their migration using an *in vitro* wound healing scratch assay, and subsequently, the conditioned medium was evaluated for their effect on human fibroblast proliferation. Subsequently, allogenic cells were intradermally injected into full-thickness, cutaneous thoracic wounds of 4 horses. Wound healing was assessed by using 3-D digital imaging and by measuring mRNA expression of pro-and anti-inflammatory markers for 30 days. Using human fibroblasts in an *in vitro* wound healing assay, we demonstrate a significantly higher healing in the presence of conditioned medium collected from proliferating MSCs than in the presence of medium containing fetal bovine serum. The *in vitro* effect of MSCs did not translate into a detectable effect *in vivo*. Nonetheless, we proved that molecularly characterized equine allogenic MSCs do not illicit an immunologic response. Investigations using MSCs derived from other sources (adipose tissue, umbilical cord), or a higher number of MSCs or a compromised animal model may be required to prove the efficacy of equine MSCs in wound healing *in vivo*.

## Introduction

Regenerative biological therapies using mesenchymal stem cells are being used extensively to treat horses for injuries. Bone marrow-derived mesenchymal stem cells or mesenchymal stromal cells (BM-MSCs) are self-renewing, expandable *in vitro*, and able to differentiate into cellular lineages, each capable of producing various types of cells, including adipocytes, osteoblasts, chondrocytes, and skin cells (1–5).

Wound healing is a dynamic process involving complex interactions between many cellular and biochemical events. Paracrine signaling results in the release of soluble factors, including growth factors, cytokines, and chemokines that promote the many biological events required for healing, such as cellular migration and proliferation, deposition of extracellular matrix, angiogenesis, and remodeling (5–8). The release of these factors from fibroblasts, inflammatory cells, skin progenitor cells, and fat- and bone marrow-derived MSCs, are crucial to these biological events (4, 6, 8–11). Several mechanisms by which MSCs exert a positive effect on wound healing have been identified. These include the enhancement of angiogenesis by secretion of pro-angiogenic factors and the differentiation into endothelial cells and/or pericytes, M2 macrophages polarization, the recruitment of endogenous stem/progenitor cells, extracellular matrix production and remodeling, and immunosuppressive effects. (12).

Studies of the effects of MSCs transplanted into wounds of human patients and experimentally created wounds of other species, including rodents, rabbits, goats, and dogs, have demonstrated the therapeutic effects of MSCs in improving dermal regeneration and healing (4, 6, 8, 9, 13–16). The reparative effect of MSCs is brought about by paracrine signaling, which results in release of biologically active molecules that affect cellular migration and proliferation, and in survival of cells surrounding the injured area (8, 11). Javazon et al. demonstrated that directly applying BM-MSCs to cutaneous wounds of diabetic mice resulted in significantly improved epithelialization, angiogenesis, and formation of granulation tissue (10). Kim et al. documented that MSC-treated wounds of dogs healed more rapidly with increased cellular proliferation, collagen synthesis, and angiogenesis than did control wounds (15). Arno et al. showed that MSCs derived from human Warton's Jelly promoted wound healing in mice by up-regulating the expression of genes involved in neovascularization, re-epithelialization, and fibroproliferation in MSC-treated fibroblasts (8).

Even though the pathophysiological mechanisms of equine wound repair, and the effects of platelet-rich plasma on equine wound healing have been reported (17, 18), reports demonstrating the effect of equine BM-MSCs on experimentally created wounds of horses are lacking. We hypothesized that eBM-MSCs have the potential to speed healing of wounds of horses, and that we could demonstrate this potential *in vitro* and *in vivo*. We hypothesized that injecting molecularly-tested, allogenic, eBM-MSCs into the periphery of a cutaneous wound would not trigger an adverse effect and would enhance epithelialization and wound contraction primarily through paracrine signaling. To prove our hypotheses, eBM-MSCs cultured from a 4-year-old, mixed-breed gelding and previously characterized with respect to their proliferation, expression of stem-cell markers, and *in vitro* capacity for tri-lineage differentiation were used (19, 20). *In vitro* scratch assays were performed to assess the effect of conditioned medium (CM) collected from eBM-MSCs on the migration and proliferation of human skin primary fibroblasts. Finally, eBM-MSCs were applied to experimentally-created, full-thickness, cutaneous thoracic wounds of 4 adult horses to evaluate the wound healing response *in vivo*.

## Materials and methods

### Animals

All experiments were carried out using institution approved protocols. Three female American Quarter horses and one, mixed-breed female horse, 12–23 years old, were used as recipients of the eBM-MSCs. All had no current or past health problems. All were housed and maintained under the same conditions, and each was confined to an individual stall.

### Allogenic eBM-MSCs

All procedures were performed as described previously (19, 20). Allogenic eBM-MSCs from one 4-year-old, mixed breed horse, which was negative for Equine infectious anemia, equine parvovirus, equine pegivirus, non-primate hepacivirus, and Theiler's diseases associated virus, were used. Cryobanked MSCs, previously characterized by their proliferation, expression of mesenchymal stem cell surface markers and *in vitro* trilineage differentiation potential were used. Cryopreserved cells were thawed rapidly in a 37°C water bath and were washed with prewarmed phenol red-free, antibiotic - free, and FBS-free media. Cells were collected by centrifugation at 200 g at room temperature for 10 min. Cells were suspended in Dulbecco's Modified Eagle Medium (DMEM F12), and incubated at 37°C in presence of DMEM F12 + 10% fetal bovine serum + 1% penicillin/streptomycin mixture. The incubator was maintained at 5% CO_2_ and 95% atmospheric air.

Equine BM-MSCs were expanded and cells from passages 4–5 were used in the *in vitro* scratch assays and for implantation into the wounds *in vivo*.

Total RNA was extracted from biopsy samples from each horse at 3, 7, 15, and 30 days after treatment. using an RNeasy Mini RNA kit^1^ according to the manufacturer's instructions and as described earlier (19). Messenger RNA expressions of pro- and anti-inflammatory targets, including, TGFβ, IGF, IL1β, TNFα, and IL8, were measured using quantitative PCR. SYBR green-based absolute blue qPCR mix^2^ with100-pM concentrations of commercially synthesized, forward and reverse primers^2^ were used. PCR conditions and the primer sequences were as described earlier (21, 22).

All qPCRs were run on the Agilent Mx3005P, and data were analyzed using MxPro analysis software^3^. Delta Ct was calculated for each sample after it was normalized with the Ct value of equine 18S RNA. Using a cDNA blank, samples with a Ct value of ≥30 were given an arbitrary expression of zero.

### Preparation of conditioned medium (cm)

Equine BM-MSCs from the allogenic donor were seeded at a cellular density of about 5000 cells/cm^2^, and the CM was collected after 48 h.

### Human foreskin fibroblasts (HFF-1)

Commercially obtained, normal human fibroblasts (HFF-1)^4^ were grown in DMEM high glucose medium^5^ containing 10% fetal bovine serum and 1% penicillin/streptomycin at 37°C, with 5% CO_2_. Passage 7–8 HFF cells were seeded at a cellular density of about 5000 cells/cm^2^, and the CM was collected after 48 h.

### Proliferation and migration of HFF

Migration of HFFs was demonstrated *in vitro* using a wound healing assay. Human fibroblasts were seeded at density of 20,000 cells/cm^2^ to form a monolayer of cells of about 80% confluency. One scratch/sample/well was made with a 200 μL pipette tip, to ensure that a gap of 400–500 μM was created in each well. Cellular debris was washed with Hank's Balanced Salt Solution^2^. Cells were then maintained in the presence of eBMMSC-CM, HFF-CM, or FBS-containing growth medium. Cells incubated in the presence of 0% eBMMSC-CM (100% FBS), 10, 25, 50, and 75% eBMMSC-CM (90–25% FBS), and 100% eBMMSC-CM (0% FBS), and HFF-CM were tested.

*In vitro* migration in the presence of freshly collected CM was also compared to the migration in the presence of a thawed sample that had been frozen at −80°C. Each assay was repeated in triplicate in two independent experiments.

For gap/wound closure or healing, two randomly selected points along each wound were identified, and the horizontal distance between the two wound edges was measured using black and white phase-contrast microscopy. Measurements were made at 16, 20, 24, 40, and 44 h, post-wounding.

The HFF proliferation rate in presence of eBMMSC-CM was assessed at 2, 4, and 7 days using the CellTiter 96^®^ Aqueous Non-Radioactive (MTS) assay^6^ according to the manufacturer's instructions. The optical density of the complex formed between cells and the MTS reagent was measured on a microplate fluorescence reader^7^ at 490 nm. Medium without cells was used as a blank. A graph of sample absorbance corrected with the blank vs. days of proliferation was generated, and data were analyzed. HFF growth medium and 100% FBS-containing MSC growth medium were used as controls.

### *In vivo* equine cutaneous wound healing model

Under sedation, two, 4-cm diameter and two, 2-cm diameter wounds were created aseptically on each side of the thorax of each recipient horse. The 4-cm diameter wounds were created at the 14th and 10th intercostal spaces on a line parallel to the ground at the level of the ventral aspect of the tuber coxae. The 2-cm diameter wounds were created at the 14th and 10th intercostal spaces, 4–6 cm ventral to the 4-cm diameter wounds. Skin at the site of wounding was desensitized with 2% mepivacaine HCl^2^. A shallow, circular cutaneous skin incision was created using a stainless steel, 4-cm or 2-cm punch. The incision was extended to subcutaneous tissue with a scalpel blade, and skin within each incision was sharply excised and designated as the day 0 sample. Sterile gauze pads were applied to each wound, and the wounds were covered with a sterile, combine bandage, which was secured to the thorax with a commercially available abdominal bandage^8^.

### Subcutaneous implantation of allogenic eBM-MSCs

Two days post-wounding, 2 × 10^6^ eBM-MSCs suspended in 1 mL sterile, isotonic saline solution were injected subcutaneously at 4 equidistant sites on each wound. The untreated wounds served as controls and were injected with the vehicle only. Wounds were dressed and bandaged as described above.

### 3-D imaging for wound measurements

All 4-cm diameter wounds were photographed immediately after wound creation (time 0), and at each bandage change. Images were imported into a computer software program linked to the 3-D camera^9^, and data were analyzed as described earlier (23). The percentage wound healing was calculated by measuring the wound area at days 3, 7, 15 and 30 post treatment. Changes in areas at each time point were compared to the areas measured at day 0, and are finally reported as the % change.

### Skin biopsies

The 2-cm diameter wounds were biopsied to collect samples for RNA isolation and qPCR. Each biopsy was obtained from a different site on each wound.

### Statistical analyses

All qPCR data were analyzed using mixed model analysis, with the gene expression ratios as the dependent variables, treatment as the between-subject fixed independent factor, time as the within-subject independent factor, and horse nested within treatment as the random factor. SAS procedure, PROC MIXED in SAS/STAT^10^ was used to conduct the analyses. Similarly, all measurements for the *in vitro* wound healing assays and the *in vivo* wound images were compared using 2-way ANOVA. Statistical significance was set to *P* < 0.05. All graphs are generated using GraphPad Prism 9.

## Results

### Equine BMMSC-CM promotes wound healing *in vitro*

**Figure 1:**
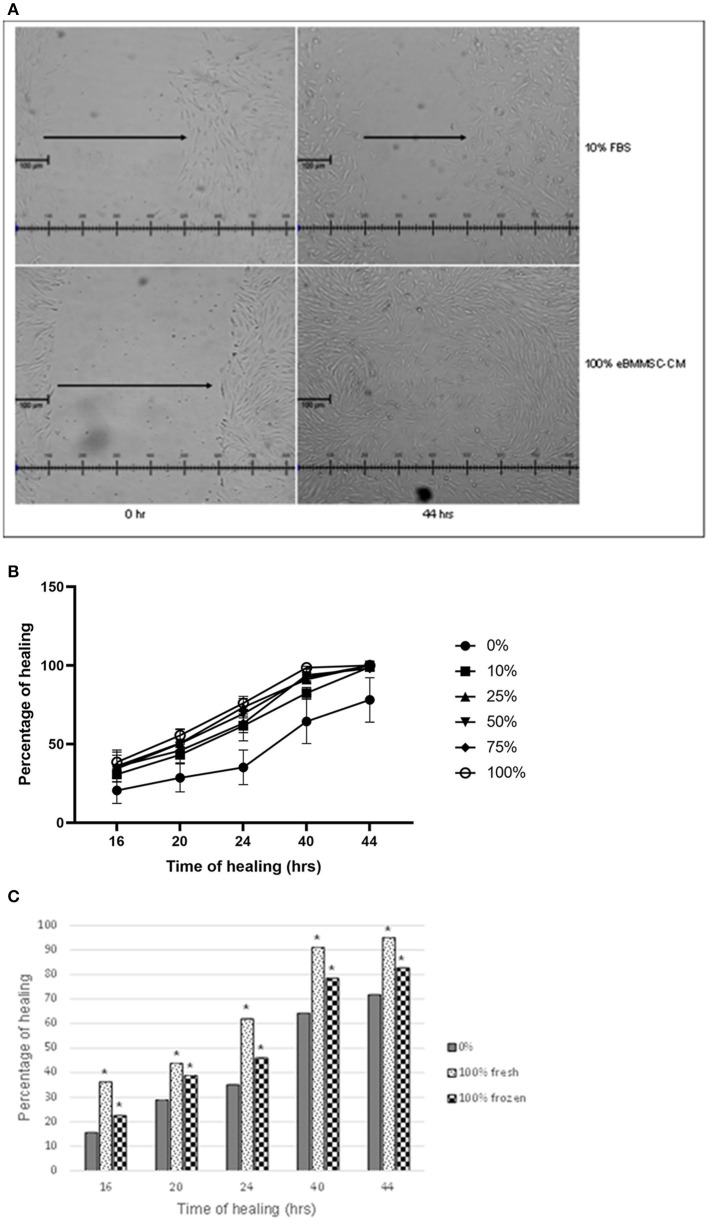
**(A)** Effect of varying eBMMSC-CM concentrations. 400–500 μm gaps were created in a monolayer of HFFs, and were treated with varying concentrations of eBMMSC-CM for 16–44 h. Gap closure was measured at each time point and data is reported as the percentage healing at a specific point relative to time 0. Results are shown as the mean ± SD of quadruplicates within a representative of two independent experiments. *P < 0.05 is considered significant. **(B)** Effect of eBMMSC-CM on HFF migration. 400–500 μm gaps were created in a monolayer of HFFs, and were treated with growth medium containing 10% FBS (Top) or 100% eBMMSC-CM (Bottom), and gap closure was evaluated using phase contrast microscopy. Representative images of quadruplicates within a representative of two independent experiments are shown. **(C)** *In vitro* wound healing assay to compare frozen and freshly isolated eBMMSC-CM. 400–500 μm gaps were created in a monolayer of HFFs, and were treated with either freshly isolated 100% eBMMSC-CM or 100% eBMMSC-CM stored in −80^0^C. Wound Gap closure was measured at each time point and data is reported as the percentage healing at a specific point relative to time 0. Results are shown as the mean ± SD of quadruplicates within a representative of two independent experiments. *P < 0.05 is considered significant.

To assess the effect of eBM-MSCs on wound healing, an *in vitro* wound healing assay was performed, wherein the HFF migration was assessed in the presence of eBMMSC-CM for 16–44 h. In the first experiment, the effect of wound closure was evaluated using a range of concentrations of the conditioned media, and wound healing was assessed in presence of 0, 10, 25, 50, 75, and 100% eBMMSC-CM (Figures 1A,B). Significant HFF migration treated with 100% eBMMSC-CM resulted in gap closure as early as 16 h (*P* < 0.05), whereas no gap closure was observed in HFFs treated with growth medium containing 10% FBS even after 44 h post-wounding (Figure 1A).

Even though all doses of eBMMSC-CM showed healing, significant effects were consistently observed at all time-points with 100% eBMMSC-CM. Figure 1B represents microscopic images comparing the wound healing in presence of medium containing 10% FBS with 100% eBMMSC-CM. All measurements were compared to measurements at time 0 and then expressed as percentage healing relative to the wound's original size.

Next, wound healing in presence of 100% freshly isolated eBMMSC-CM was compared with that in the presence of eBMMSC-CM stored in −80°C. This experiment was carried out to mimic a clinical condition in which freshly expanded or cryopreserved allogenic MSCs could be provided for immediate use. As demonstrated in Figure 1C, significant gap closure was observed at all time-points in presence of both fresh and frozen CM. Note that freshly collected CM provided a relatively higher healing percentage, which was not statistically significant.

### Equine BMMSC-CM promote the proliferation of HFF *in vitro*

**Figure 2:**
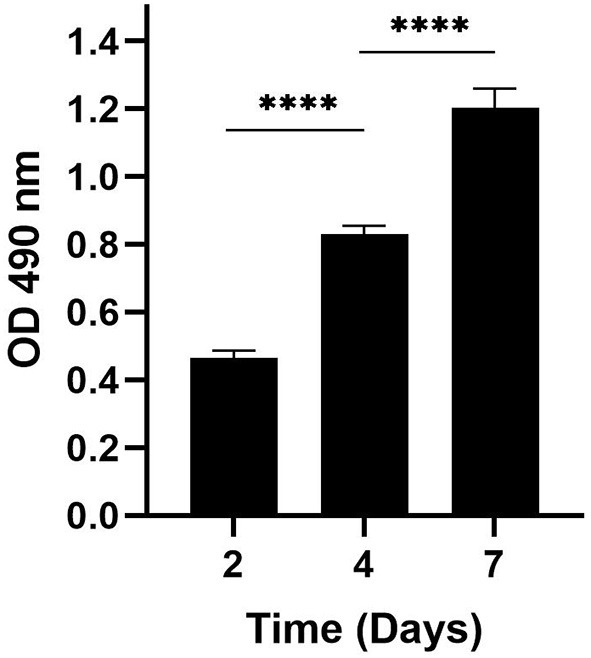
Measurement of proliferation rate of HFFs. MTS assay was used to demonstrate the proliferation of HFFs in presence of 100% eBMMSC-CM over a period of 7 days. The results represent the mean ± SD with *n* = 3 for each bar. ****p < 0.0001.

Because proliferation of fibroblasts is an important aspect of wound healing, we next determined whether the increased incidence of wound closure in HFFs is also accompanied by an increase in enhanced proliferation of fibroblasts treated with eBMMSC-CM. As illustrated in Figure 2, HFFs were cultured for 7 days in presence of freshly isolated 100% eBMMSCs-CM, and the HFF proliferation was measured using the MTS assay. Cell proliferation linearly increased 3-fold within 7 days. No cell death was observed during this time.

### Lack of immunologic response to allogenic eBM-MSCs

As determined by visible and subjective clinical analyses, all horses remained clinically normal throughout the study period. Appetite, body weight, activity, body temperature, and heart and respiratory rates were always within normal limits. Healing in all wounds progressed without visible signs of infection or immunogenic reaction suggesting that the allogenic MSCs could be used safely *in vivo*.

### Effect of equine BMMSC-CM on wound healing *in vivo*

**Figure 3:**
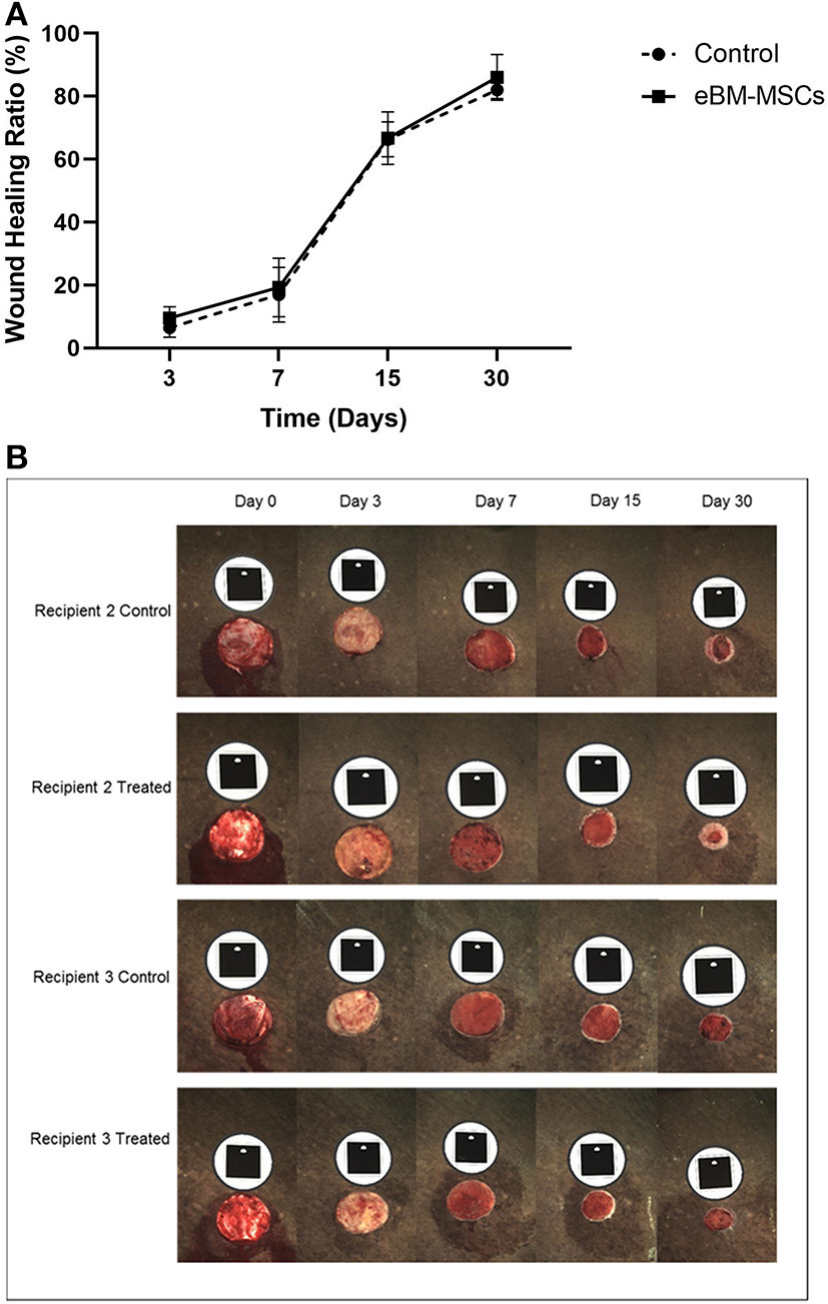
Wound healing in recipient horses. **(A)** Graph showing percentage changes in wound healing in all horses as a function of time. The wound area is measured in mm^2^ at time 0, and subsequently at days 3, 7, 15 and 30. The area at each time point is then compared to the area at time 0. Finally, the % wound healing is expressed and plotted graphically. **(B)** Representative digital images of the 4 cm diameter wounds in the recipients 2 and 3 at various time points are shown. The black tag above each wound is the marker and is recognized by the camera to ensure a consistent and reproducible angle when the image is taken. Note the significant amount of epithelialized region of the wound in the Recipient 2 treated panel.

Next, we assessed if the *in vitro* effects observed with eBMMSC-CM could be translated to an *in vivo* wound model. All 4-cm diameter wound images were analyzed using a trace-area function in the software. All measurements were recorded in mm^2^ (23). Using the digital images, we measured the Healing rate (mm^2^/day) = (previous total area – subsequent inner area) ÷ number of days between the two time-points (Figure 3A). The healing rate was used to assess the combined effect of epithelialization and contraction because these two processes occur simultaneously and could not be segregated. Epithelialization was observed subjectively in all groups only after day 15.

As seen in the representative pictures from one recipient horse, subjectively, eBMMSC-treated wounds appeared to show a faster healing rate with increased epithelialization compared to controls (Figure 3B). These changes were observed in 2 out of 4 recipients indicating horse-to-horse variation. As a result, when data from all the horses were taken together, none of the measurements obtained above were significantly different between control and stem cell-treated wounds.

### *In vivo* mRNA expression of pro– and anti–inflammatory markers

**Figure 4:**
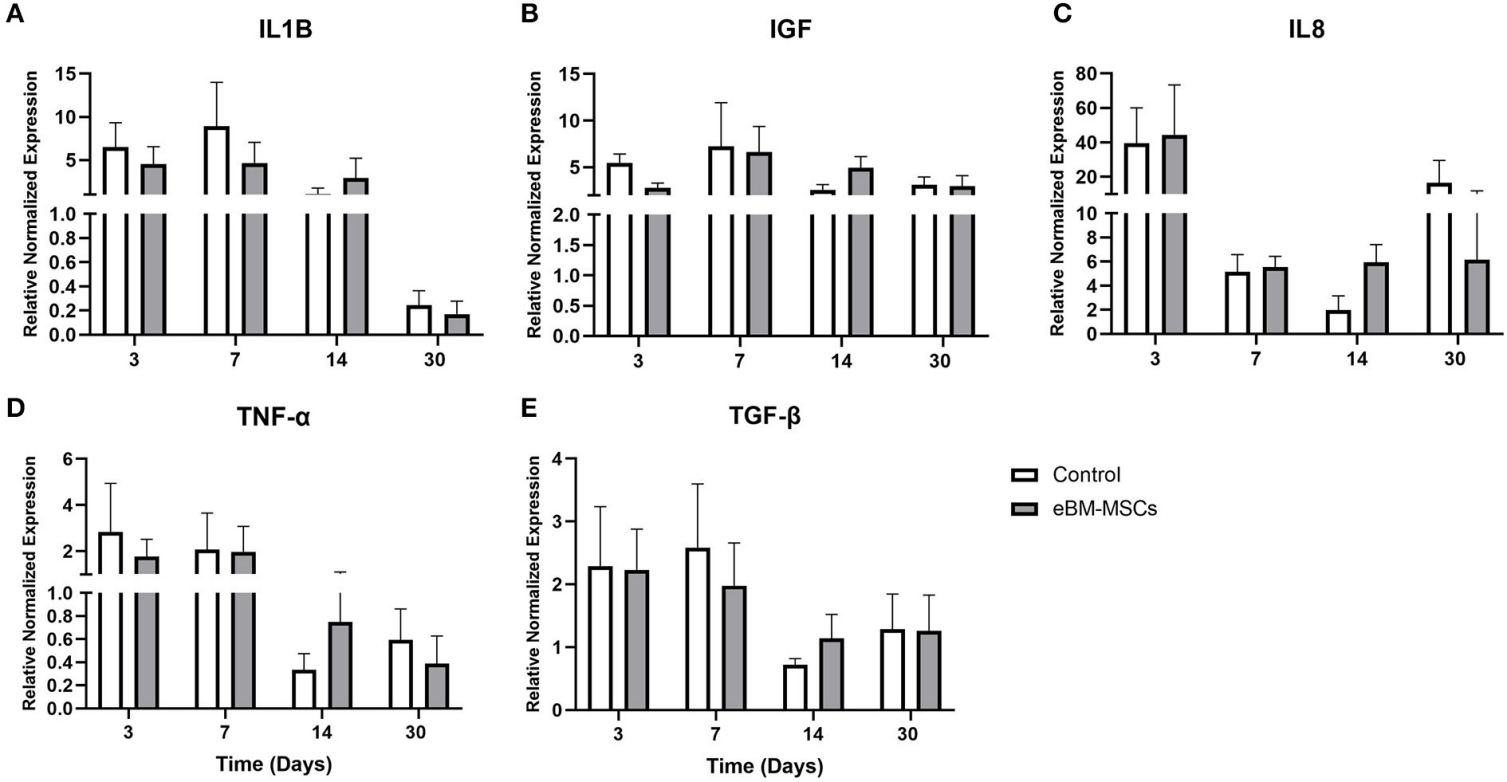
Real time PCR of the pro- and anti-inflammatory markers. The expression profiles of ILβ **(A)**, IGF **(B)**, IL8 **(C)**, TNFα **(D)** and TGFβ **(E)** were generated at days 3, 7, 15 and 30 post therapy. Changes in expression for each mRNA was normalized with the Ct values obtained for 18S RNA and are plotted as Delta Ct in arbitrary units (AU). *P* < 0.05 was set as significant.

Different phases of wound healing are accompanied by changes in cytokines, and therefore, we assessed whether there were any eBM-MSC-mediated modulations of mRNA expression of TGFβ, IGF, IL1β, TNFα, and IL8, pre- and post-treatment. Real-time PCR profiles of each of the markers were assessed at days 3, 7, 15, and 30 post-treatment. Samples of skin collected when the wounds were created (day 0) were used as pretreated samples. Normalized delta Ct values of each mRNA obtained at days 3,7,15, and 30 were compared day 0 to assess these changes (Figure 4). Results demonstrated no significant changes in expression between control and treated groups during the entire study.

## Discussion

Bone-marrow-derived MSCs are multipotent cells that have the potential to differentiate into multiple lineages, including lineages capable of producing adipocytes, osteocytes, chondrocytes, neurons, skeletal muscle, and endothelial cells (1–5). Adult MSCs are ideal to use in cellular-based therapies because of the relative ease of techniques for isolation and expansion and because of their low immunogenicity.

Using the minimal set of standard criteria (24, 25), we have identified an allogenic donor from which MSCs were generated and cryopreserved and used in this study. Because BM-MSCs obtained from different organisms, including horses, are immunomodulatory and lack the MHC II, they should not illicit an immunologic response when implanted into the tissues of another animal. With this knowledge, we were confident that we could safely implant eBM-MSCs from the allogenic donor into the experimentally created wounds of the 4 horses used in this study. As expected, none of the recipient horses showed signs of immunological response to the allogenic cells.

Cutaneous wound healing is a complex process primarily involving the migration and proliferation of fibroblalsts. We used an immortalized, commercially available HFF-cell line, and fibroblasts cultured served to assess dermal fibroblastic response to injury *in vitro*. We assessed the effect of CM derived from healthy, proliferating eBM-MSCs on proliferation of HFFs and subsequently cultured the human HFFs and created a “wound” on the monolayer and assessed the paracrine effect that the eBM-MSCs had on closure of that wound.

The *in vitro* analyses revealed that eBMMSC-CM enhances both the migration and proliferation of human fibroblasts, suggesting that eBM-MSCs may secrete a factor or factors necessary for healing, thus supporting their paracrine function. This agrees with other studies (26, 27). For instance, *in vitro* studies have shown that conditioned medium from fat-derived MSCs alone accelerates wound healing in mice due to the paracrine effect of the medium. Pro-angiogenic factors, such as vascular endothelial growth factor (VEGF), are secreted from MSCs, and these factors have been suggested to be one of the pro-angiogenic mechanisms by which fat-derived MSCs accelerate healing *in vivo* (26, 27). Similarly, promotion of proliferation and migration of mouse keratinocytes *in vivo* by conditioned medium of BM-MSCs has been demonstrated (28).

In our study, the significant effect of eBM-MSCs on wound healing observed *in vitro* did not translate consistently *in vivo*, even though digital imaging showed an increased healing in the BMMSC-treated wounds in 2 out of 4 recipients. We have several hypotheses to explain this discrepancy. One important factor to consider is the source of MSCs. Fat-derived equine MSCs might prove to be better than eBM-MSCs at healing of equine wounds. Liu et al. demonstrated that human, fat-derived MSCs had a more substantial effect on the cutaneous wound healing of mice than did MSCs derived from the amnion and bone marrow. Histological evaluation showed enhanced epithelialization only in the group treated with fat-derived MSCs (29).

Secondly, using MSCs to speed wound healing may be beneficial only when healing is impaired or compromised. Mesenchymal stem cells might prove beneficial when applied to non-healing and chronic wounds, such as those of elderly human patients, diabetic patients, patients with large injuries, and patients receiving glucocorticosteroid therapy. Wounds of these patients have impaired production of cytokines and reduced angiogenesis (30–33). The experimentally created wounds on the horses' thorax in this study were acute, and the horses were systemically normal. Furthermore, in this model, since the wounds were not chronic, the paracrine effect of the eBM-MSCs may have been inconsequential, because the growth factors involved in wound healing may have been abundant and functional, making those supplied by the MSCs redundant. MSCs might prove useful when applied to chronic wounds depleted of functional or containing non-functional growth factors. Because wounds on the distal portion of the limb of horses heal more slowly than those on the thorax (34), a limb wound model might be considered compromised and may be more likely to demonstrate positive effects of exogenous MSCs. The disadvantage of using an equine limb wound model, however, is exuberant granulation tissue formation which may confound the results. In a pilot study using 2 horses, we found that control wounds and eBMMSC-treated wounds on the distal portion of the limb filled with exuberant granulation tissue. The results were difficult to interpret when measures to control exuberant granulation tissue formation were instituted (data not shown).

Finally, the viability and the sustained presence of eBM-MSCs at the site of implantation also raise some doubt about the efficacy of MSCs in wound healing in this model.

Latest tissue engineering approaches emphasize the application of scaffolds in wound healing (35–37). Even though a variety of natural and synthetic material-based scaffolds are being fabricated, appropriate knowledge of the physicochemical properties of the biomaterials and scaffolds is needed. At the same time, one has to evaluate the interaction between endogenous progenitor and exogenously delivered stem cells with the scaffolds and hence, even though there is potential, practical skin scaffold materials remain to be developed. Challenges exist that we have to circumvent before translating this technology to skin tissue engineering.

The *in vivo* portion of this study was a pilot experiment to assess whether the paracrine effect of eBM-MSCs observed *in vitro* could be translated in a relatively simple wound model, and hence, a small sample number of horses were used. As demonstrated in Figure 3, objective measurements showed that the wounds of 2 of the 4 treated horses had improved healing, but, this positive effect was not significant when data from all 4 horses was analyzed collectively. Nonetheless, our data clearly supports the use of molecularly–tested equine allogenic eBM-MSCs to be a potential source of cells for the treatment of cutaneous wounds without any adverse effects. Even though there are published reports that demonstrate that mesenchymal stem cells do have the potential to heal equine cutaneous wounds (38–40), finding an optimal source of MSCs, finding an optimal number of cells to use, time and the route at which the therapy should be applied, and priming MSCs by exposing them to bioactive scaffolds are some of the aspects of regenerative medicine, which should be considered for a consistent and reproducible response.

In summary, we present data showing that eBM-MSCs enhance the proliferation and paracrine function of cutaneous fibroblasts. This *in vitro* effect induced by BM-MSCs, however, could not be replicated consistently, *in vivo* using our model. The influence of the source of equine MSCs for *in vivo* application to wounds requires more investigation, and the model used for *in vivo* assessment and delivery of MSCs may need to be improved.

The concentration and the quality of RNA was evaluated using the RNA 6000 Nano Kit and the 2100 Bioanalyzer system as per the manufacturer's recommendations (Agilent Technologies, Santa Clara, CA). An electropherogram showed intact 28S and 18S RNA bands and confirmed high quality RNA with RIN values > 9.0.

## Data availability statement

The original contributions presented in the study are included in the article/supplementary materials, further inquiries can be directed to the corresponding authors.

## Ethics statement

The animal study was reviewed and approved by University of Tennessee, Institutional Animal and Care Committee.

## Author contributions

JS and MD were responsible for all *in vivo* and *in vitro* experimental planning, execution, data analysis and interpretation, respectively. MC carried out all *in vivo* experiments and data analysis. SS carried out *in vitro* wound assays and assisted MC in the animal studies. HE and LA carried out the fibroblast proliferation and real time PCR assays. All authors proof read the manuscript and gave their consent for publication.

## Funding

This work was supported by the Center of Excellence in Livestock Diseases and Human Health, University of Tennessee, Knoxville.

## Conflict of interest

The authors declare that the research was conducted in the absence of any commercial or financial relationships that could be construed as a potential conflict of interest.

## Publisher's note

All claims expressed in this article are solely those of the authors and do not necessarily represent those of their affiliated organizations, or those of the publisher, the editors and the reviewers. Any product that may be evaluated in this article, or claim that may be made by its manufacturer, is not guaranteed or endorsed by the publisher.

## Footnotes

^1^Qiagen, Valencia, California, USA.

^2^Fisher Scientific, Pittsburgh, Pennsylvania, USA.

^3^Agilent Technologies, Santa Clara, California, USA.

^4^ATCC, Manassas, VA, USA.

^5^Carl Zeiss Inc., Thornwood, New York, New York, USA microscope.

^6^Promega Inc., Madison, Wisconsin, USA.

^7^BioTeck, Winooski, Vermont, USA.

^8^Wire 2 Wire Vet Products, LLC, Lexington, KY, USA.

^9^Eykona Technologies Ltd., Fuel3D Inc., Greenville, NC, USA.

^10^SAS Institute Inc., Cary, NC, USA.
